# Diversity and Fermentation Products of Xylose-Utilizing Yeasts Isolated from Buffalo Feces in Thailand

**DOI:** 10.1264/jsme2.ME13023

**Published:** 2013-09-04

**Authors:** Wanlapa Lorliam, Ancharida Akaracharanya, Motofumi Suzuki, Moriya Ohkuma, Somboon Tanasupawat

**Affiliations:** 1 Department of Biochemistry and Microbiology, Faculty of Pharmaceutical Sciences, Chulalongkorn University, Bangkok 10330, Thailand; 2 Department of Microbiology, Faculty of Science, Chulalongkorn University, Bangkok 10330, Thailand; 3 Microbe Division, Japan Collection of Microorganisms (JCM), RIKEN BioResource Center, 3–1–1 Koyadai, Tsukuba, Ibaraki 305–0074, Japan

**Keywords:** Xylose-utilizing yeast, Buffalo feces, *Candida*, *Geotrichum*, *Trichosporon*

## Abstract

Twenty-eight xylose-utilizing yeast strains were isolated by enrichment culture from 11 samples of feces from the rectum of Murrah buffalo and Swamp buffalo in Thailand. On the basis of their morphological and biochemical characteristics, including sequence analysis of the D1/D2 region of the large-subunit ribosomal RNA gene (LSU rDNA), they were identified as *Candida tropicalis* (designated as Group I, 11 isolates), *Candida parasilosis* (Group II, 2 isolates), *Candida mengyuniae* (Group III, 2 isolates), *Sporopachydermia lactativora* (Group IV, 2 isolates), *Geotrichum* sp. (Group V, 5 isolates) and *Trichosporon asahii* (Group VI, 6 isolates). All isolates utilized xylose as the sole carbon source but 27 isolates could ferment xylose to ethanol (0.006–0.602 g L^−1^) and 21 isolates could ferment xylose to xylitol (0.19–22.84 g L^−1^). *Candida tropicalis* isolates produced the highest yield of xylitol (74.80%). Their ability to convert xylose to xylitol and ethanol ranged from 15.06 g L^−1^ to 22.84 g L^−1^ xylitol and 0.110 g L^−1^ to 0.602 g L^−1^ ethanol, respectively.

Lignocellulosic biomass varies among plant species but generally consists of around 25% lignin and around 75% carbohydrate polymers (cellulose and hemicellulose). It is the largest known renewable carbon source (2, 22). D-xylose, a pentose sugar and the main hydrolysis product of lignocellulosic biomass, is the second most abundant fermentable material (16). Xylose fermentation is challenging because only a few microorganisms, such as bacteria and yeasts, can readily ferment xylose. Xylose-fermenting yeasts such as *Candida shehatae*, *Pachysolen tannophilus*, *Brettanomyces naardenensis*, *Candida tenuis*, *Scheffersomyces segobiensis*, *Candida lyxosophila*, *Candida intermedia*, *Candida jeffriesii*, *Spathaspora passalidarum*, *Spathaspora arborariae*, *Candida prachuapepsis* and *Scheffersomyces stipitis* strains are ethanol producers from xylose (5, 15, 30, 31). In addition, xylitol is a polyol (sugar alcohol) obtained from the reduction of xylose by strains of *Candia boidinii*, *Candida guilliermondii*, *Candida maltosa*, *Candida parapsilosis*, *Candida tropicalis*, *Debaryomyces hansenii*, *Pachysolen tannophilus*, *Meyerozyma caribbica*, *Pichia miso*, *Issatchenkia* sp., and *Clavispora* sp. (11, 33, 35, 40, 41). Xylitol is an alternative sweetener, as sweet as sucrose, equivalent to 2.4 kcal g^−1^ and laxative nature (145 J g^−1^ caloric content) (33). It has recently drawn the attention of food and drink manufacturers due to its low caloric value and thus the possibility of its use to reduce and control weight, leading to applications as a sweetener in many products and in the pharmaceutical industry (11). Currently, xylose-fermenting yeasts have been isolated from soil, the gut of beetles, wood, rooting wood, estuarine water from a mangrove forest, the gut of coleopteran insects, fruits, tree bark, etc. (5, 7, 30, 35, 36, 37). In Thailand, the distribution of xylose-utilizing yeasts in herbivore animal feces, especially in buffalo feces, has not yet been reported. Buffaloes are also called water buffaloes. There are two broad categories of buffaloes, river buffaloes and swamp buffaloes. The Murrah buffalo (river buffalo) is the best buffalo breed for milk production (26). Swamp buffaloes are used for multiple purposes in Thailand. They are fed on foliage, crop residues, agro-industrial by-products and non-conventional feed resources (48). Therefore, this study deals with the first attempt to isolate xylose-utilizing yeasts from feces from the rectum of two types of buffaloes, Murrah and Swamp buffaloes, in Thailand, to identify them at a specific level based on their phenotypic characteristics and sequence analysis of the D1/D2 region of the large-subunit ribosomal RNA gene (LSU rDNA D1/D2) and to determine their xylose-fermentation products.

## Materials and Methods

### Collection of samples and the isolation and maintenance of the yeast isolates

Buffalo fecal samples were collected at Murrah Farm in December 2009, in Chachoengsao province, Thailand. Eleven fecal samples were taken directly from each rectum of Murrah and Swamp buffaloes of different ages (Table 1). Each 0.5 g sample was enriched in a tube containing 10 mL YX medium (0.67% yeast nitrogen base, 5% D-xylose) supplemented with 200 mg L^−1^ chloramphenicol and 0.25% sodium propionate. The enrichment samples were incubated at 30°C for 3–10 days and were spread on YX agar for their isolation. The number of detected colonies was less than 49/sample. Representative yeast colonies were selected based on colonial characteristics, purified using a single colony isolation method and maintained on a YM agar slant (0.3% yeast extract, 0.3% malt extract, 0.5% peptone, 1% glucose and 1.5% agar) at 4°C or in freezing tubes containing YM broth supplemented with 10% glycerol at −80°C.

### Phenotypic characterization

Morphological characteristics were examined according to Yarrow (49) and Kurtzman *et al.* (18). Formation of true- and pseudo-hyphae were monitored in cornmeal agar at 25°C until 7 days and ascospore production was examined on cornmeal agar, 5% malt extract and YM agar until 2 months. For physiological characteristics, Yeast identification system ID 32 C (bioMérieux, Marcy l’Etoile, France) was used according to the manufacturer’s instructions. The kit allows the evaluation of the assimilation of 30 carbon sources for clinical isolates of pathogenic yeasts. Test strips were incubated at 30°C for 48 h (24 to 48 h is recommended).

### DNA sequence and phylogenetic analysis

A loopful of yeast cells was suspended in 100 μL lysis buffer in a 1.5 mL Eppendorf tube (34) and was boiled in a water bath or metal block bath for 15 min. After boiling, 100 μL of 2.5 M potassium acetate (pH 7.5) was added, placed on ice for 1 h, and centrifuged at 14,000 rpm for 5 min. The supernatant was extracted twice with 100 μL chloroform/isoamyl alcohol (24:1 [v/v]) and DNA in the upper layer was precipitated with ethanol, dried and dissolved in 30 μL MilliQ water. The D1/D2 domain of the large subunit ribosomal RNA gene (LSU rDNA D1/D2) was amplified by polymerase chain reaction (PCR) with primers NL1 (5′-GCATATCAATAAGCGGAGGAAAAG-3′) and NL4 (5′-GGTCCGTGTTTCAAGACGG-3′) (32). The PCR condition was performed according to the methods described for the amplification of 26S rDNA D1/D2 domain (20). The PCR product was checked by agarose gel electrophoresis and purified using a QIAquick purification kit (Qiagen, Tokyo, Japan). The purified PCR product was sequenced using the BigDye Terminator v.3.1 Cycle Sequencing RR-100 kit and an ABI Model 3130xl DNA Analyzer (Applied Biosystems, Foster City, CA, USA) following the manufacturer’s instructions. The sequencing reactions were performed using the external primers NL1 and NL4. Sequence data were aligned using the program Chromas Pro software (Technelysium Pty, South Brisbane, Australia). The sequences were compared with available sequence data using a BLASTN search (1) and were aligned with sequences of related species retrieved from GenBank using the multiple alignment program CLUSTAL_X version 1.8 (45). A phylogenetic tree was constructed from the evolutionary distance data with maximum composite likelihood correction using the neighbor-joining method (38). The topology of the phylogenetic tree was tested by performing bootstrap re-sampling from 1,000 replicates (10). Sequences determined in this study were deposited in the DDBJ gene databank (Shizuoka, Japan) and their accession numbers are shown in Table 1.

### Production of ethanol and xylitol from xylose

The fermentation of D-xylose was tested by cultivation in YP medium (1% yeast extract and 2% peptone), adjusted to pH 5.5 with HCl and supplemented with 6% D-xylose on 50 mL YP medium in a 250 mL Erlenmeyer flask, shaken on a rotary shaker at 200 rpm at 30°C until 24 h. *S. stipitis* JCM 10742^T^ (Japan Collection of Microorganisms, RIKEN BioResource Center, Tsukuba, Japan) was used as a reference strain.

### Analysis of substrates and products

Fermentation broth was centrifuged at 8,000 rpm for 10 min and the supernatants were used to determine the ethanol concentration using a gas chromatograph (GC-9A; Shimadzu, Kyoto, Japan), the concentration of xylose and xylitol concentration using HPLC with a Lichrospher100 NH2 (5 μm) column of 250 mm × 4 mm (Merck, Germany) and detected with an evaporative light scattering detector (Alltech, Illinois, USA). Acetonitrile/water (91:1) was used as the mobile phase at a flow rate of 1.5 mL min^−1^.

## Results and Discussion

**Fig. 1:**
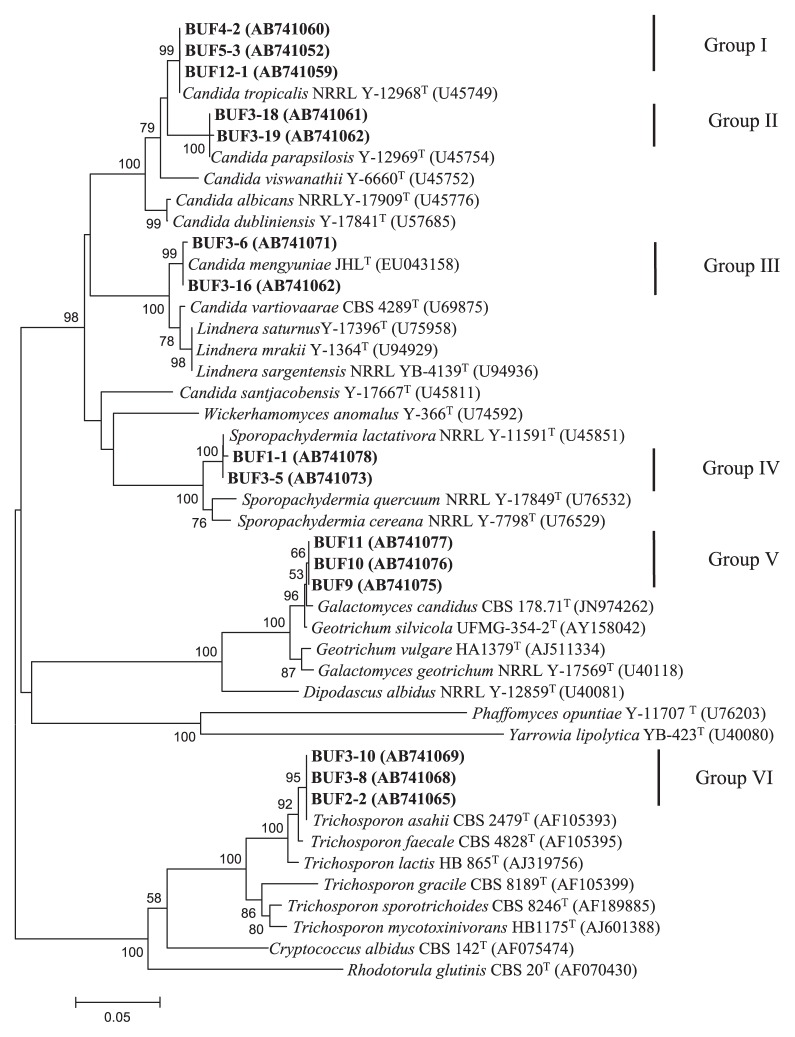
Phylogenetic tree of xylose assimilation yeasts constructed by the neighbor-joining method based on the D1/D2 domain of LSU rRNA gene sequences. Numbers represent the percentages from 1,000 replicate bootstrap resamplings (frequency <50% is not shown). Bar indicates *K*nuc.

This study presents the first characterization of xylose-utilizing yeast associated with six samples of Murrah buffalo feces and five samples of Swamp buffalo feces that were enriched in YX medium. The cell densities of yeasts in Murrah buffalo feces were higher than in Swamp buffalo feces. However, the xylose-utilizing yeasts obtained from each buffalo fecal sample were less than 100 colony-forming units. The characterization of 28 isolates is summarized in Tables 1 and 2, and Fig. 1.

### Identification of isolates

Twenty-eight xylose-utilizing yeast isolates were divided into six groups based on their phenotypic characteristics and the sequences of D1/D2 region analyses (Tables 1 and 2, and Fig. 1), designated as Group I to VI. Five groups were assigned to five ascomycetous yeast species, *C. tropicalis* (Group I, 11 isolates), *C. parapsilosis* (Group II, 2 isolates), *Candida mengyuniae* (Group III, 2 isolates), *Sporopachydermia lactativora* (Group IV, 2 isolates) and *Geotrichum* sp. (Group V, 5 isolates) related to *Geotrichum candidum*. Only one group of basidiomycetous yeast utilizing xylose was assigned to *Trichosporon asahii* in the genus *Trichosporon* (Group VI, 6 isolates).

Group I contained 11 isolates. All the isolates assimilated cellobiose, galactose, gluconate and glucosamine, maltose, α-methyl-d-glucoside, mannitol, melezitose, trehalose, sucrose and xylose (Table 2). They had almost the same phenotypic characteristics as *C. tropicalis* NRRL Y-12968^T^ (21). The isolates had identical sequences in the nucleotide sequence of the D1/D2 domain of the 26S rRNA gene. The representative three isolates were located within the cluster of *C. tropicalis* in the phylogenetic tree in Fig. 1. Therefore, they were identified as *C. tropicalis*.

Group II contained 2 isolates, BUF3-18 and BUF3-19. Two isolates assimilated l-arabinose, galactose, gluconate, glucosamine, maltose, mannitol, melezitose, α-methyl-d-glucoside, sucrose, trehalose (latent) and xylose but did not assimilate ribose (Table 2). Their phenotypic characteristics were similar to *C. parapsilosis* Y-12969^T^ (4). The isolates were closely related to *C. parapsilosis* Y-12969^T^ based on the identical nucleotide sequence of the D1/D2 domain of the 26S rRNA gene (Table 1, Fig. 1). Therefore, they were identified as *C. parapsilosis*.

Group III contained 2 isolates, BUF3-6 and BUF3-16. They showed 99.8% similarity of the nucleotide sequence of the D1/D2 domain of the 26S rRNA gene to *C. mengyuniae* JHL^T^ (Table 1) but some of their phenotypic characteristics were different from this type strain (6). These isolates assimilated dl-lactate, maltose and melezitose but did not assimilate galactose, gluconate, lactose and melibiose (Table 2). The nucleotide sequence of other genes should be confirmed for this group.

Group IV contained 2 isolates, BUF1-1 and BUF3-5, The isolates assimilated glycerol, glucose, lactose and xylose. These isolates could not assimilate inositol but *S. lactativora* NRRL Y-11591^T^ could (37). The isolates showed 99.2–99.4% similarity of the nucleotide sequence of the D1/D2 domain of the 26S rRNA gene to *S. lactativora* NRRL Y-11591^T^ (Table 1, Fig. 1), and were identified as *S. lactativora*.

**Fig. 2:**
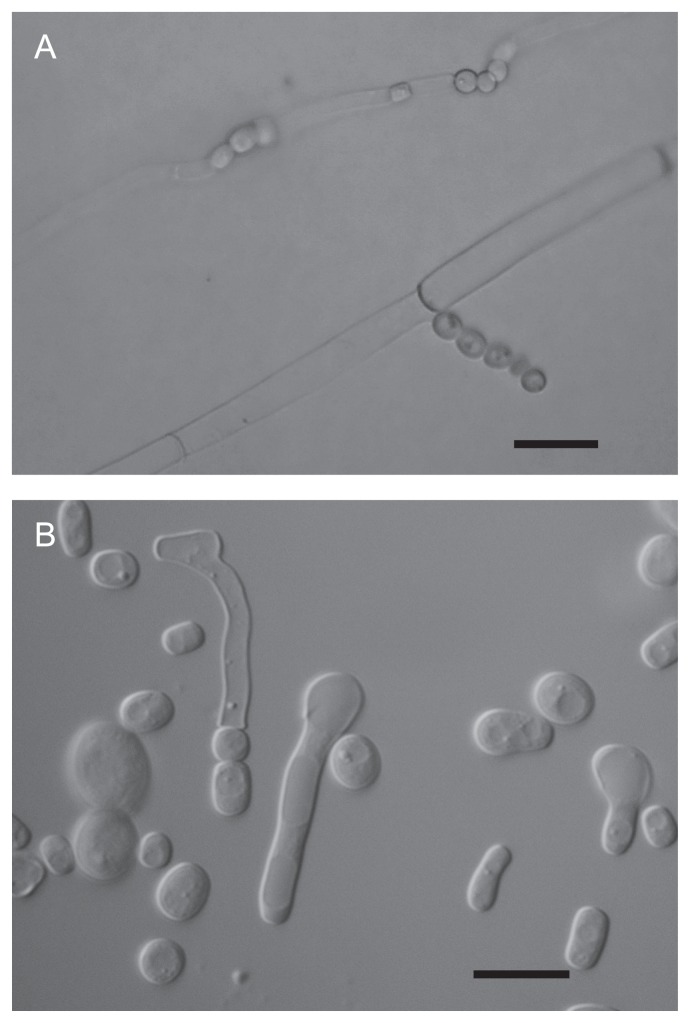
Morphology of *Geotrichum* sp. BUF11 observed by light microscopy. True-hyphae formed on corn meal agar at 25°C for 5 days (A). Vegetative cell grown on 5% malt extract agar at 25°C for 3 days (B). Bar indicates 5 μm.

Group V contained 5 isolates, BUF7, BUF8, BUF9, BUF10 and BUF11. The isolates showed 97–99% similarity of the nucleotide sequence of the D1/D2 domain of the 26S rRNA gene to that of *Geotrichum candidum* or its teleomorph, *Galactomyces candidus*. In the phylogenetic tree, all these isolates were clustered with their nearest phylogenetic neighbors in the genus *Geotrichum* and its teleomorphic genus *Galactomyces*, but were separated from the other *Geotrichum* species (Fig. 1). From pairwise comparison of these sequences with *G. candidum*, 4–11 nucleotide (nt) substitutions were found in 537 nucleotides. From these data, this group may include divergent populations within a species or different species from *G. candidum*. Kurtzman and Robnett (20) compared the divergence among ascomycetous strain pairs with previously determined nuclear DNA reassociation values and it appeared that different species differed by 6 or more nucleotides among the 500–600 nucleotides of the D1/D2 domain. Moreover, the isolates showed 81.4% similarity of the nucleotide sequence of the internal transcribed spacer region (ITS) to that of the type strain of *G. candidum* (data not shown). The morphological characteristics of these isolates, such as the formation of white, hairy, usually dry colonies, true hyphae, arthroconidia and blastoconidia (Fig. 2), supported that they were members of the genus *Geotrichum* (8). They assimilated galactose, mannitol, glucose and sorbose and grew on cycloheximide medium at 37°C. However, this data were insufficient for designation to different taxa. Smith *et al.* (42) assigned strains in the genus *Georichum* to one taxon on the basis of phenotypic criteria and mating reactions. According to ‘The Yeasts: a taxonomic study, 5^th^ edition (2011)’, many species and genera have been reclassified by molecular taxonomic methodology using such as the sequence divergence of the large subunit (LSU) and small subunit (SSU) rDNA, actin-1 gene, RNA polymerase II (RPB1 and RPB2) gene, translation elongation factor-1α (TEF1α) and mitochondrial regions (19). Consequently, taking these into consideration, we assigned this Group V as *Geotrichum* sp.

Group VI contained 6 isolates. The isolates assimilated gluconate, glucosamine, dl-lactate, melezitose, l-rhamnose, and sucrose but did not assimilate glycerol or inositol. The phenotypic characteristics were almost the same as the type strain (28) (Table 2). The isolates had an identical nucleotide sequence of D1/D2 to *T. asahii* CBS 2479^T^ (Table 1). They were basidiomycetous yeast; therefore, they were identified as *T. asahii*.

### Yeasts associated with buffalo rectum feces

From Table 1, the two types of buffalo feces, Murrah buffalo and Swamp buffalo (*Bubalus bulalis*), appeared to have different microbiota (or microflora) types of yeast species. In Swamp buffalo feces, only 2 species, *C. tropicalis* and *Geotrichum* sp., were found. In each individual feces in young to old animals, *C. tropicalis* or *Geotrichum* sp. or both was found. On the other hand, in Murrah buffalo feces, *C. tropicalis* and *Geotrichum* sp. were found only in old individuals. In fecal samples from young Murrah buffalos aged 1, 2 and 2.2 months, *S. lactativora*, *T. asahii* or both were found. In addition to these two species, *C. mengyuniae* and *Candida parapsilosis* were detected in feces from Murrah buffalos aged 2 months. Almost all yeast strains isolated in this study were so-called opportunistic pathogens, such as *T. asahii* and *C. parapsilosis*.

Swamp buffalo are very well adapted to hot and humid climates as well as marshy lands (26). Murrah buffalo are highly sensitive to solar radiation, and the poorly evolved body thermoregulation of buffaloes is compensated for by their wallowing activity in fresh water bodies (39). From these reports it was shown that Murrah buffalo may have weaker health than Swamp buffalo. Therefore, the Murrah buffalo in this research might have been infected with yeast pathogens.

Previous studies have concentrated on the search for yeasts in the gastrointestinal tract (GIT) of vertebrates with a focus on farm animals, and researchers have detected various ascomycetous and basidiomycetous yeasts, chiefly representing the genera *Candida*, *Trichosporon*, *Pichia*, *Rhodutorula*, *Debaryomyces*, *Kluyveromyces* and *Saccharomyces*. (46, 47). *C. tropicalis* has been isolated from a variety of animal GIT, such as cows, sheep, pigs and beetles (46). Suzuki *et al.* (44) also reported the occurrence of *C. tropicalis* in a number of fruits in Thailand. However, this yeast is one of the more frequently encountered clinical yeast species after *C. albicans* (29). Similarly, *C. parapsilosis* is considered to be the second most important opportunistic pathogenic yeast (4, 23). *T. asahii* have a medical association, being commonly found as the causative agent of disseminated mycoses in patients with impaired immunity, and occasionally as the cause of human or animal white piedra (28). *S. lactativora* strains appear to be cosmopolitan in distribution, such as in soil, fermented food, decaying agriculture residue and bird feces in Antarctica (9, 24, 31), and they could grow at high temperatures of 37°C or more (37).

Species of the genera *Geotrichum* and *Galactomyces*, such as *Galactomyces geotrichum*, *Geotrichum silvicola*, and *Geotrichum* sp., have been isolated from animal GIT on farms (8, 46) and are distributed worldwide.

*C. mengyuniae*, a sulfonylurea herbicide-resistant yeast, was isolated from fields often soaked with discharge from metsulfuron-methyl manufacturing facilities (6). The occurrence of *C. mengyuniae* in Murrah buffalo of 2 months old may show that this buffalo is eating food contaminated with herbicide.

In addition, xylose-utilizing yeast from gut of passalid beetles, *S. stipitis*, *S. segobiensis*, *C. shehatae* and *Candia ergatensis*, and yeasts from the digestive tract and feces of animals, *C. tropicalis*, *S. lactativora* and *T. asahii*, have been reported (25, 43, 46). *Kazachstania slooffiae*, *Candida glabrata*, *I. orientalis* (a synonym of *Pichia kudriavzevii*), *Pichia fermentans*, *T. ashaii* and *C. tropicalis* strains in the gut of piglets kept under experimental conditions, and *Kazachstania slooffiae*, *G. geotrichum*, *Candida catenulata*, *Trichosporon coremiiforme* and *T. asahii* in the gut of piglets on a commercial farm were studied (46). In Thailand, xylose-utilizing yeasts, *C. tropicalis*, *C. albicans*, *Pichia terricola*, *S. lactativora*, *Trichosporon mycotoxinivorans* and *Zygoascus meyerae*, have been isolated from herbivore feces, such as from elephants, goats, giraffes, kangaroos, horses, cows and zebras (25). In comparison to this study, we found *C. tropicalis*, *C. parapsilosis*, *C. mengyuniae*, *S. lactativora*, *Geotrichum* sp. and *T. asahii* in buffalo feces.

## Ethanol and xylitol fermentation

Twenty-eight isolates in this study utilized xylose as their sole carbon source. All of these isolates could ferment xylose to ethanol (0.006–0.602 g L^−1^) and 21 isolates could ferment xylose to xylitol (0.19–22.84 g L^−1^) using YX medium (20% xylose and 0.67% yeast nitrogen base) over 24 h in a 50 mL flask (Table 3). Fermentation results revealed that all yeasts tested were able to consume d-xylose, with consumption rates ranging from 44.60% to 100% in 24 h, except that BUF3-10 consumed 8.63%. This strain had low consumption of xylose but could produce ethanol and xylitol. *C. tropicalis* strains produced the highest xylitol and ethanol production among the strains (Table 3). Their xylitol production was in the range of 15.06 g L^−1^ to 22.84 g L^−1^ and their ethanol production was 0.110 to 0.602 g L^−1^ in medium containing 60 g L^−1^ d-xylose at 24 h. *Candida tropicalis* showed the highest xylitol productivity (Q_xy_=0.63 g L^−1^ h^−1^ to 0.95 g L^−1^ h^−1^), with minimum and maximum %Y_xy/s_ values of 29.30% to 74.84%. Strain BUF 9-4 had the highest %Y_xy/s_ value because of its low xylose consumption compared with the same strains and high xylitol productivity (20.02 g L^−1^). This group prefers to produce xylitol more than ethanol. *Candida* yeast in particular has been studied with regards to its biotechnological application in the production of xylitol. Barbosa *et al.* (3) reported that xylitol yield was as high as 0.77 g g^−1^ or 77% for *C. guilliermondii* and 0.85 g g^−1^ or 85% for *C. tropicalis. C. parapsilosis* showed xylose consumption of around 58% and only produced ethanol of 0.006 to 0.049 g L^−1^, which was very low. So, yeast strains used xylose as their sole carbon source for growth only. Also, strains assigned as *Geotrichum* sp. had xylose consumption of 61.20% to 75.30% but very poor ethanol and xylitol production. Strains of *C. mengyuniae*, *S. lactativora* and *T. asahii* produced little ethanol and xylitol production. However, their xylitol production was more than ethanol production (Table 3). *S. stipitis* JCM 10742^T^ was used as a positive control. This xylose-fermenting yeast produced high ethanol, (%Y_e/s_=28.5) but showed low xylitol production (%Y_xy/s_=1) (data not shown).

Recently, there have been many attempts to isolate xylose-utilizing yeast that produces high ethanol or xylitol. *S. arborariae* strain from rotting wood in Brazil was reported to produce ethanol (0.50 g g^−1^ xylose) (5) and *Candida saraburiensis* from decaying agriculture residue produced ethanol (3.1–3.6 g L^−1^ at 72 h) (31). *Spathaspora passalidrum* from the gut of passalid beetles could produce ethanol (0.4 g g^−1^ xylose) (13), and *C. guilliermondii* Xu280, *C. maltosa* Xu316 and strain YS54 could produce xylitol 0.73 g g^−1^, 0.70 g g^−1^ and 0.58 g g^−1^, respectively (12, 35). Our present study was the first screening of xylose-utilizing yeasts from buffalo feces; therefore, further study is required on xylose fermentation based on several factors that would affect the biosynthesis of xylitol and ethanol production, such as initial xylose concentration, pH, inoculums, culture media, aeration rate and redox imbalance (14, 17, 27, 33).

## Conclusion

In this study, xylose-utilizing yeasts distributed in feces from the rectum of buffalo were investigated for the first time. The results showed that they contained members of the genera *Candida*, *Sporopachydermia*, *Trichosporon* and *Geotrichum*. Only two yeast species, *Candida tropicalis* and *Geotrichum* sp., were isolated from young and old Swamp buffalo feces as well as from old Murrah buffalo feces. Moreover, *T. asahii* and *C. parapsilosis* isolates were found in young Murrah buffalo feces. In addition, *C. mengyuniae*, known as a sulfonylurea herbicide-resistant yeast, was isolated from the same young Murrah buffalo feces. All strains of xylose-fermenting yeasts utilized xylose as their sole carbon source. Some strains could ferment xylose to ethanol and xylitol. In particular, *C. tropicalis* showed the highest yield of xylitol production.

## Figures and Tables

**Table 1:** Source, isolation and identification of xylose-utilizing yeasts

Samples	Isolate no.	Group	%Similarity	Identification	aAccession number
Murrah buffalo (1 month old)	BUF2-1	VI	100	*T. asahii*	AB741064
	BUF2-2	VI	100	*T. asahii*	AB741065
	BUF2-4	VI	100	*T. asahii*	AB741066
Murrah buffalo (2 months old)	BUF3-10	VI	100	*T. asahii*	AB741069
	BUF3-16	III	99.8	*C. mengyuniae*	AB741072
	BUF3-18	II	100	*C. parapsilosis*	AB741061
	BUF3-19	II	100	*C. parapsilosis*	AB741062
	BUF3-3	VI	100	*T. asahii*	AB741067
	BUF3-5	IV	99.4	*S. lactativora*	AB741063
	BUF3-6	III	99.8	*C. mengyuniae*	AB741071
	BUF3-8	VI	100	*T. asahii*	AB741068
Murrah buffalo (2.2 months old)	BUF1-1	IV	99.2	*S. lactativora*	AB741078
Murrah buffalo (2.52 years old)	BUF9	V	99	*Geotrichum* sp.	AB741075
	BUF9-4	I	100	*C. tropicalis*	AB741058
	BUF9-2	I	100	*C. tropicalis*	AB741056
	BUF9-5	I	100	*C. tropicalis*	AB741057
Murrah buffalo (2.58 years old)	BUF8	V	99	*Geotrichum* sp.	AB741074
	BUF8-1	I	100	*C. tropicalis*	AB741054
	BUF8-2	I	100	*C. tropicalis*	AB741055
Murrah buffalo (2.65 years old)	BUF10	V	99	*Geotrichum* sp.	AB741076
Swamp buffalo (2 months old)	BUF7	V	99	*Geotrichum* sp.	AB741073
	BUF7-1	I	100	*C. tropicalis*	AB741053
Swamp buffalo (3 months old)	BUF5-3	I	100	*C. tropicalis*	AB741052
Swamp buffalo (3.8 months old)	BUF4-2	I	100	*C. tropicalis*	AB741060
	BUF4-3	I	100	*C. tropicalis*	AB741050
	BUF4-4	I	100	*C. tropicalis*	AB741051
Swamp buffalo (4.33 years old)	BUF11	V	97	*Geotrichum* sp.	AB741077
Swamp buffalo (5.1 years old)	BUF12-1	I	100	*C. tropicalis*	AB741059

a Accession number: LSU rDNA D1/D2 sequences determined in this study and deposited in the DDBJ gene databank in Japan.

**Table 2:** Differential characteristics of the isolates in Group I, II, III, IV, V, VI and related type strains

Characteristics	1	2^a^	3	4^a^	5	6^b^	7	8^c^	9	10^d^	11	12^e^
Assimilation of												
l-Arabinose	−	−	+	+	−	−	−	−	−	−	+	+
Cellobiose	+	v	−	−	v	−	−	−	−	−	+	+
Erythritol	−	−	−	−	−	−	−	−	−	−	+	v
Galactose	+	+	+	+	−	+	−	−	+	+	+	+
Glycerol	−	v	+	+	+	+	+	+	+	+	−	v
Gluconate	+	v	+	v	−	nd	−	−	−	−	+	−
Glucosamine	+	v	+	v	−	−	−	−	−	−	+	v
Glucose	+	+	+	+	+	+	+	+	+	+	+	+
Inositol	−	−	−	−	−	−	−	s	−	−	−	v
dl-Lactate	−	v	−	−	+	nd	−	−	l	+	+	v
Lactose	−	−	−	−	−	nd	+	v	−	−	+	+
Maltose	+	+	+	+	+	−	−	−	−	−	+	+
d-Mannitol	+	+	+	+	+	+	−	−	+	+	v	v
Melezitose	+	v	+	+	+	−	−	−	−	−	+	v
Melibiose	−	−	−	−	−	+	−	−	−	−	−	−
α-Methyl-d-glucoside	+	v	+	+	v	+	−	−	−	−	+	+
Raffinose	−	−	−	−	+	+	−	−	−	−	−	−
l-Rhamnose	−	−	−	−	−	−	−	−	−	−	+	
d-Ribose	−	v	−	v	−	−	−	−	−	−	+	+
Sucrose	+	v	+	+	+	+	−	−	−	−	+	v
Trehalose	+	+	l	+	+	+	−	−	−	−	v	+
d-Xylose	+	+	+	+	+	+	+	+	+	+	+	+

1, Group I (11 isolates); 2, *C. tropicalis*; 3, Group II (2 isolates); 4, *C. parapsilosis*; 5, Group III (2 isolates); 6, *C. mengyuniae*; 7, Group IV (2 isolates); 8, *S. lactativora*; 9, Group V (5 isolates); 10, *G. candidum*; 11, Group VI (6 isolates); 12, *T. asahii*.

+, positive; −, negative; l, latent (longer than 7 days), s, slow; w, weak; v, variable; n, no data.

a Data from Lachance *et al.* (21).

b Data from Chen *et al.* (6).

c Data from Rodrigues *et al.* (37).

d Data from de Hoog and Smith (8).

e Data from Molnar *et al.* (28).

**Table 3:** Xylose fermentation parameters of yeast cultivated in d-xylose culture medium assays under aerobic conditions for 24 h

Yeast species	Yeast strain	D-Xylose consumption (%)^a^	Ethanol concentration (g L^−1^)	%Ye/s^b^	Xylitol concentration (g L^−1^)	Q_xy_ (g L^−1^ h^−1^)^c^	%Yxy/s^d^
*C. tropicalis*	BUF5-3	97.80	0.329	0.34	22.34	0.93	38.10
*C. tropicalis*	BUF4-2	98.15	0.169	0.17	22.84	0.95	38.80
*C. tropicalis*	BUF4-3	65.53	0.225	0.34	21.19	0.88	53.90
*C. tropicalis*	BUF4-4	98.57	0.178	0.18	20.15	0.84	34.10
*C. tropicalis*	BUF7-1	98.64	0.297	0.30	21.35	0.89	36.10
*C. tropicalis*	BUF8-1	53.15	0.333	0.63	15.06	0.63	47.20
*C. tropicalis*	BUF8-2	96.92	0.602	0.62	17.04	0.71	29.30
*C. tropicalis*	BUF9-2	95.80	0.474	0.49	19.69	0.82	34.30
*C. tropicalis*	BUF9-4	44.60	0.452	1.01	20.02	0.83	74.80
*C. tropicalis*	BUF9-5	99.72	0.133	0.13	20.12	0.84	33.60
*C. tropicalis*	BUF12-1	100.0	0.110	0.11	21.62	0.90	36.00
*C. parapsilosis*	BUF3-18	58.02	0.006	0.01	0.00	0.00	0.00
*C. parapsilosis*	BUF3-19	58.00	0.049	0.09	0.00	0.00	0.00
*C. mengyuniae*	BUF3-6	68.59	0.060	0.09	2.59	0.11	6.30
*C. mengyuniae*	BUF3-16	61.60	0.160	0.26	0.9	0.04	2.40
*S. lactativora*	BUF1-1	51.57	0.071	0.14	0.00	0.00	0.00
*S. lactativora*	BUF3-5	80.22	0.000	0.00	3.68	0.15	7.70
*Geotrichum* sp.	BUF7	75.30	0.048	0.06	0.19	0.01	0.40
*Geotrichum* sp.	BUF8	65.71	0.074	0.11	0.4	0.02	1.00
*Geotrichum* sp.	BUF9	68.04	0.060	0.09	0.00	0.00	0.00
*Geotrichum* sp.	BUF10	61.20	0.113	0.18	0.00	0.00	0.00
*Geotrichum* sp.	BUF11	70.85	0.039	0.06	0.00	0.00	0.00
*T, asahii*	BUF2-1	67.29	0.042	0.06	0.00	0.00	0.00
*T. asahii*	BUF2-2	80.88	0.130	1.16	0.81	0.03	1.70
*T. asahii*	BUF2-4	65.78	0.029	0.04	1.12	0.05	2.80
*T. asahii*	BUF3-10	8.63	0.028	0.32	0.34	0.01	6.50
*T. asahii*	BUF3-8	89.19	0.006	0.01	0.88	0.04	0.02
*T. asahii*	BUF3-3	58.90	0.040	0.07	0.26	0.01	0.70

a d-Xylose consumption (%)—percentage of initial d-xylose consumed;

b Ye/s-ethanol yield (g ethanol g^−1^ d-xylose consumed);

c Qxy-volumetric xylitol production rate (g L^−1^ h^−1^);

d Yxy/l-xylitol yield (g xylitol g^−1^ d-xylose consumed).
